# Cervical sagittal alignment after different anterior discectomy procedures for single-level cervical degenerative disc disease: randomized controlled trial

**DOI:** 10.1007/s00701-017-3312-z

**Published:** 2017-09-08

**Authors:** Roland D. Donk, Hisse Arnts, Wim I. M. Verhagen, Hans Groenewoud, Andre Verbeek, Ronald H. M. A. Bartels

**Affiliations:** 1 Department of Orthopedic Surgery, Via Sana Clinics, Hoogveldseweg 1, 5451 AA Mill, The Netherlands; 2 Department of Neurosurgery, Radboud University Medical Center, Geert Groote Plein-zuid 10, 6525 GA Nijmegen, The Netherlands; 3 Department of Neurosurgery, Canisius Wilhelmina Ziekenhuis, Weg door Jonkerbos 100, 6532 SZ Nijmegen, The Netherlands; 4 Department of Neurology, Canisius Wilhelmina Ziekenhuis, Weg door Jonkerbos 100, 6532 SZ Nijmegen, The Netherlands; 5 Department for Health Evidence, Radboud University Medical Center, Geert Groote Plein-zuid 10, 6525 GA Nijmegen, The Netherlands

**Keywords:** Spinal alignment, Cervical, Anterior cervical discectomy, Cage, Fusion, Arthroplasty

## Abstract

**Background:**

The effect of anterior cervical discectomy without fusion (ACD), ACD with fusion by stand-alone cage (ACDF) or with arthroplasty (ACDA) on cervical sagittal alignment is not known and is the subject of this study.

**Methods:**

A total of 142 adult patients with single-level cervical disease were at random allocated to different procedures: ACD (45), ACDF (47) and ACDA (50). Upright cervical spine radiographs were obtained. Angles of the involved angle and the angle between C2 and C7 were determined.

**Results:**

After a mean follow-up of 25.4 ± 18.4 months, the angles of the involved level comparing ACD with ACDA and ACD with ACDF were different, reaching statistical significance. However, the angle between C2 and C7 did not differ between groups or between preoperative values and at follow-up.

**Conclusions:**

Irrespective of the technique used for anterior cervical discectomy for single-level degenerative disc disease, the alignment of the cervical spine is unaltered.

## Introduction

Global sagittal balance of the spine is currently a main focus of research. Several studies have been published, stressing the importance of correct sagittal balance in relation to the quality of life [15, 25, 27]. As a consequence, the attention given to cervical alignment is also increasing. Measurements such as the T1 slope and C2–C7 sagittal vertical axis (SVA) have been introduced. A good correlation between these measurements on full-spine radiographs and the “classical” measurements on sagittal cervical radiographs has been established [8].

Anterior cervical approaches to degenerative disc disease are very familiar to spine surgeons and might affect the cervical sagittal alignment. The first descriptions of anterior cervical discectomy without fusion (ACD) and ACD with fusion (ACDF) were reported almost at the same time by Hirsch [16] and Cloward [9], respectively. Local kyphosis had already been mentioned by Hirsch in his original article, as had fusion of the involved level [16]. Cloward stated that prevention of osteophytic spur formation could be prevented by fusion [9]. Kyphosis as well as prevention of spur formation might be the reason that ACDF became more popular and is considered the golden standard.

Although proper investigations about the clinical superiority of ACDF have never been performed, ACD has almost been abandoned in clinical practice. This is remarkable since ACD provides similar adequate decompression without the need for any implant [21, 24].

Cervical sagittal balance has been investigated in patients after ACDA and ACDF [13, 18, 19, 23]. However, the effect of ACDF with a stand-alone cage or ACD on cervical sagittal balance has never been evaluated or compared with ACDA. This study fills this scientific gap in the literature.

## Methods

The Ethics Committee CMO Arnhem-Nijmegen approved the trial (CMO-no. 2002/200). The study was carried out in accordance with the World Medical Association Declaration of Helsinki [12]. A single-center, randomized controlled trial was designed comparing ACD, ACDF and ACDA [5]. From October 2003 till April 2010, patients were included in the study after having signed informed consents. However, inclusion was prematurely ended after publication of a meta-analysis comparing ACDF and ACDA [6], since we could not justify continuing the trial according to the standards of Good Clinical Practice.

Patients with (1) arm pain not responding to conservative treatment and (2) that lasted longer than 10 weeks with (3) single level disc degeneration and (4) a mobile spine on dynamic lateral cervical X-rays were included in the (PROCON) trial. After informed consent, they were allocated to ACD, ACDF or ACDA. For randomization, a closed envelope method was used. A medical secretary unaware of the purpose of the study disclosed the decision. Because of radiological follow-up, neither patients nor investigators were blinded. However, the surgeons and investigators did not have a preference for any surgical method. Clinical and radiological follow-up was initially scheduled at regular intervals: 1 day postoperatively, 6 weeks, 3 months, 1 year and 2 years postoperatively. During the trial, the follow-up protocol was altered in consultation with the ethics committee and after the requests of several patients who asked whether outpatient clinical visits were necessary. They preferred completing the questionnaires at home. The protocol was changed, and patients were asked to visit the outpatient clinic preferentially till 1 year postoperatively. Afterwards, it was voluntary. If they decided to complete the questionnaires at home, they were sent to them by flat mail.

Upright cervical spine radiographs were made. Radiographs were digitized and available using Impax ES (Agfa Web 1000 5.1, Agfa-Gevaert group, Mortsel, Belgium). The Harrison posterior tangent method was used as an estimate for measuring cervical alignment [14, 27]. A positive angle resembled lordosis, whereas a negative one defined kyphosis. The curvature was also estimated using a slight modification of the method by Toyama et al. [28]. A line was drawn from the posterior and inferior part of the vertebral body of C2 to the upper posterior part of the vertebral body of C7 [7, 11]. The curvature was defined as lordotic if the posterior wall of the vertebral bodies of C3 to C6 were anterior to this line. The cervical spine was considered straight if the posterior part of the vertebral bodies C3 to C6 were on that line and kyphotic if the posterior parts of the vertebral bodies were posterior to this line. Two investigators, experienced in measuring spine angles (HA, RB), independently measured the angle of the involved levels as well as the angle between C2 and C7, both preoperatively and at the postoperative follow-up (FU) times. They also estimated the curvature of the whole cervical spine. For statistical analyses, SAS version 9.2 (SAS Institute Inc., Cary, NC, USA) was used. Inter-rater reliability was assessed by calculating Cronbach’s alpha for the measurements at the involved level and for the C2–C7 angle. For comparison of baseline characteristics, ANOVA or chi-square tests were used. Data were represented as mean ± standard deviation (minimum–maximum). When appropriate, 95% confidence intervals were provided. Statistical significance was assumed if *P* < 0.05.

## Results

**Fig. 1:**
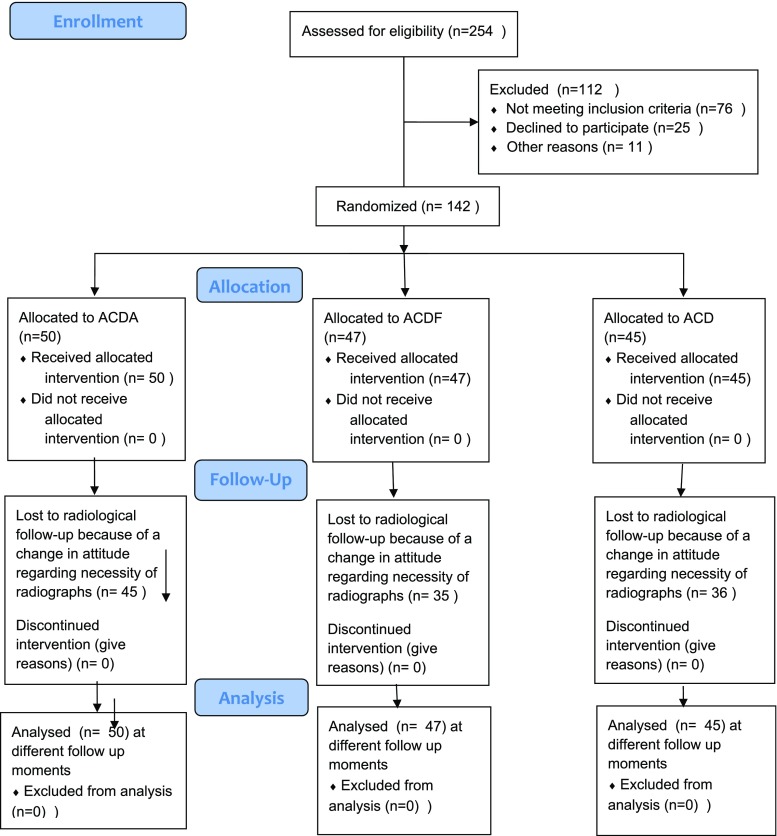
Flow chart of included patients according to Consort 2010

Of the 142 patients who were included in the study, 45 were allocated to ACD, 47 to ACDF and 50 to ACDA. The mean age of the entire group was 44.3 ± 7.0 years (18.3–59.8) and the female-to-male ratio 1:1. Baseline characteristics regarding age or gender did not statistically differ (*P* = 0.287, respectively, *P* = 0.853). The baseline characteristics for the groups are presented in Table 1. Mean radiological follow-up was 25.4 ± 18.4 months, whereas mean clinical follow-up was 9.1 ± 1.9 years (5.6–12.2). The flow chart according to Consort is represented in Fig. 1. Actual radiological follow-up differed from the follow-up protocol. This variation was due to availability of the preferences of physicians and patients. Among the three groups, there was no statistically significant difference in follow-up (*P* = 0.18). Moreover, no preoperative statistically significant differences could be detected among treatment groups in either the mean angle of the involved (affected) level or mean global sagittal (C2–C7) alignment (Table 1). The Cronbach’s alpha was 0.837 for measurements of the involved level and 0.907 for measurements of the C2–C7 angle, indicating a high inter-rater reliability. The mean values for the follow-up angles of the involved level and C2–C7 are presented in Tables 2 and 3.

**Table 1:** Distribution of mean age, gender (percentage of column), angles and total number of patients related to procedure

	ACDA	ACDF	ACD
Male	24 (48%)	25 (53.2%)	22 (48.9%)
Female	26 (52%)	22 (46.8%)	23 (51.1%)
Age, years	53.6 ± 6.9	52.2 ± 8.1	54.0 ± 6.1
Angle level involved	2.3 ± 4.6	3.2 ± 5.3	1.7 ± 5.6
C2–C7 angle	14.8 ± 13.6	12.8 ± 12.4	16.2 ± 12.6
C4–C5 level	0	2	1
C5–C6 level	21	19	26
C6–C7 level	29	26	18
Total number	50	47	45

**Table 2:** Angle in degrees at the involved level (mean ± SD) at the different follow-up (FU) times. Number of calculations (N) was also represented

	Time postoperatively (mean ± SD) (weeks)	N	ACDA	ACDF	ACD	P- value
Preoperative	N/A	138	2.3 ± 4.6	3.2 ± 5.3	1.7 ± 5.6	0.393
Directly postoperatively	N/A	140	2.5 ± 6.1	8.4 ± 5.2	−4.1 ± 05.8	.000
FU2	9.3 ± 8.6	140	1.4 ± 5.5	4.7 ± 6.1	−2.4 ± 5.5	0.000
FU3	47.0 ± 35.2	131	1.4 ± 5.5	4.7 ± 6.1	−2.4 ± 05.5	.000
FU4	134,4 ± 75.6	83	0.3 ± 6.1	3.6 ± 6.9	−2.9 ± 4.1	0.001
FU5	147.8 ± 57.3	27	1.8 ± 5.4	3.7 ± 4.3	−3.6 ± 04.2	.039

**Table 3:** Angle in degrees between C2 and C7 (mean ± SD) at the different follow-up (FU) times. Number of calculations (N) was also represented

	Time postoperatively (mean ± SD) (weeks)	N	ACDA	ACDF	ACD	P- value
Preoperative	N/A	135	14.8 ± 13.6	12.8 ± 12.4	16.2 ± 12.6	0.459
Directly postoperatively	N/A	140	12.8 ± 10.7	13.3 ± 11.4	9.8 ± 10.4	0.260
FU2	9.3 ± 8.6	139	15.0 ± 11.6	16.8 ± 10.3	13.0 ± 10.5	0.265
FU3	47.0 ± 35.2	128	14.0 ± 12.7	16.6 ± 12.8	16.5 ± 11.0	0.524
FU4	134,4 ± 75.6	83	14.0 ± 12.9	18.3 ± 13.1	17.1 ± 11.1	0.386
FU5	147.8 ± 57.3	26	14.9 ± 11.5	16.2 ± 13.5	17.3 ± 6.4	0.919

**Fig. 2:**
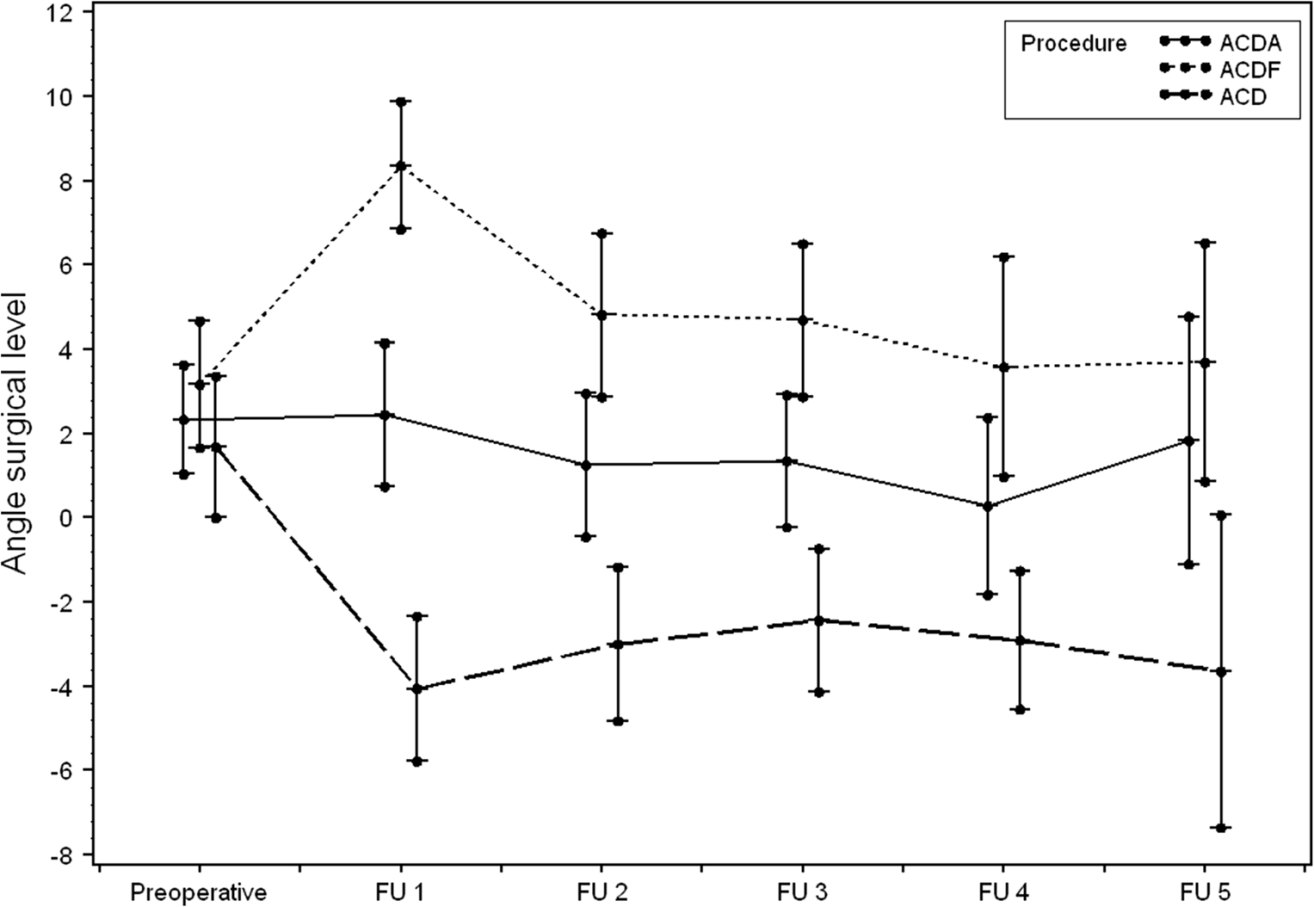
Graphs depicting the preoperative measurements of the sagittal angles at the surgical level and the values at the different follow-up times

**Fig. 3:**
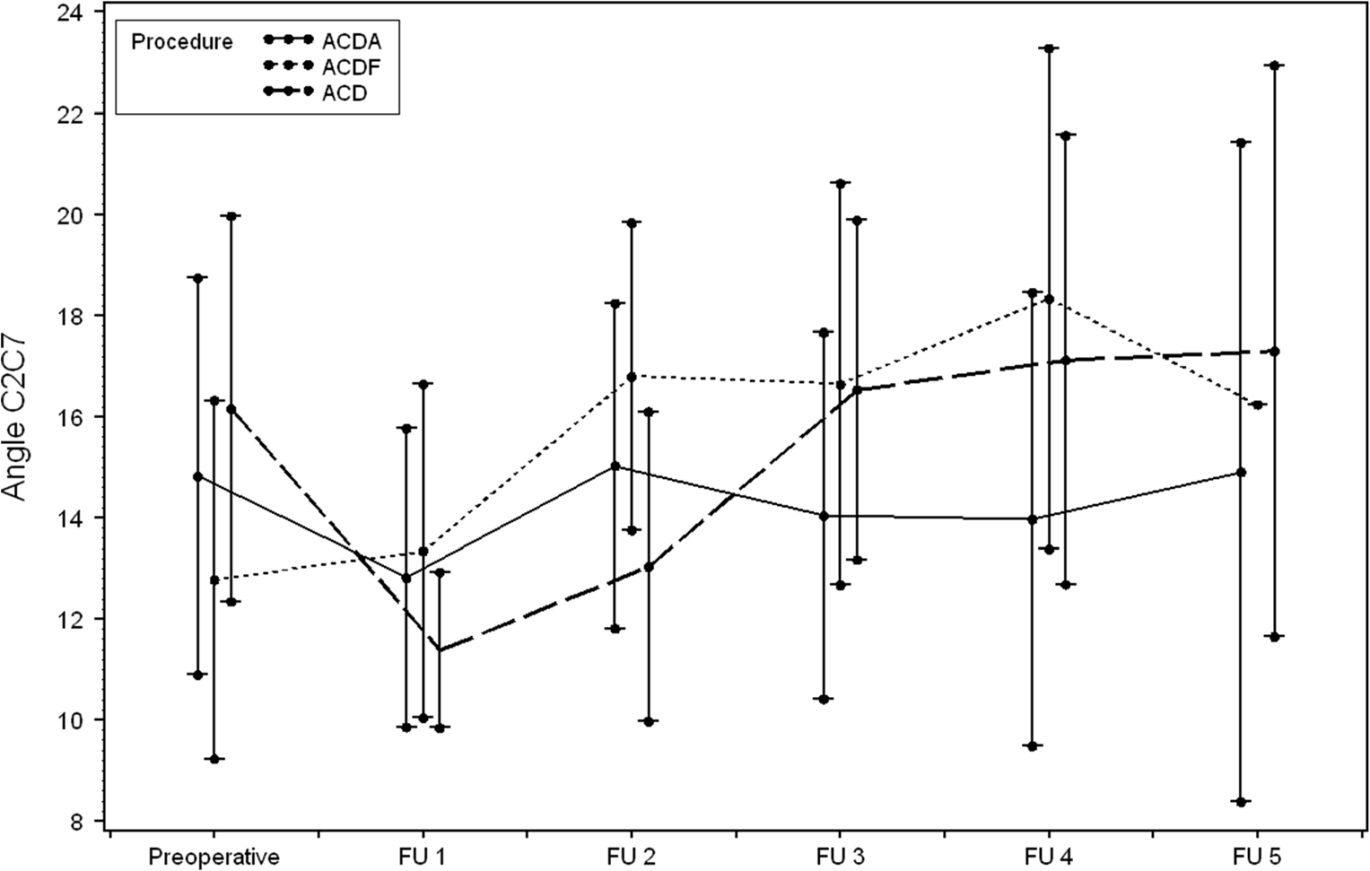
Graphs showing the preoperative measurements of the sagittal angles at C2–C7 and the values at the different follow-up times

The overall mean angles were 2.4 ± 5.2 degrees and 14.5 ± 12.9 degrees. One day postoperatively, a clear difference was observed in the angle of the involved level compared with the preoperative one. At the following follow-up times, a gradual decline to the baseline preoperative value was seen in all groups. In the ACD group, this was less prominent. Figure 2 clearly depicts that directly postoperatively (FU1: day 1 postoperatively) a statistically significant transformation is found at the involved level in patients who underwent ACD and ACDF. This was not the case for ACDA. In the ACD group, a more negative angle was found directly postoperatively, indicating the introduction of local kyphosis. However, after FU2 (9.3 ± 8.6 weeks postoperatively), changes of the angle at the involved levels did not occur anymore. Therefore, the angle measured at FU2 seemed to represent the final situation. Between FU2 and FU5 (147.8 ± 57.3 weeks postoperatively), the difference did not reach statistical significance for any of the groups. Since only 28 patients were evaluated after FU5 for reasons explained in the discussion, the 95% confidence intervals were very wide. Therefore, we compared the difference with the measurements at FU4 (134.4 ± 75.6 weeks postoperatively). The differences between FU2 and FU4 were also not statistically significant (except for the angle at the involved level for ACDA). The mean differences within each group at the successive follow-up times could be considered measurement error (Table 4). The same observation was made for the C2–C7 angle (Table 5). Between the treatment groups, differences existed when comparing the local angle of the involved level. This can be clearly seen in Fig. 2. The mean values for ACDA were 1.4 ± 5.5 degrees for ACDF 4.7 ± 6.1 and for ACD -2.4 ± 5.5 (*P* < 0.0001) at FU3 (*N* = 131). For the C2–C7 angle, statistical significance was not reached when comparing the groups (*P* = 0.305) as depicted in Fig. 3. The shape of the cervical spinal curve did not change in 111 patients during follow-up. In 31 patients the shape did alter (Table 6), but none became kyphotic. The ultimate shape of the curvature was not dependent upon the used technique, but (statistically significant) upon the preoperative shape (*P* < 0.001).

**Table 4:** Differences in angles in degrees at the involved level [mean (95% CI)] for the different follow-up (FU) times (PREOP: preoperative; POSTOP: 1 day postoperatively)

	ACDA	ACDF	ACD
POSTOP-PREOP	0.1 (−1.7; 2.0)	5.2 (3.8; 6.6)	−5.2 (−7.3; −3.1)
FU2-POSTOP	−1.2 (−2.2; −0.2)	−3.3 (−4.7; −1.8)	0.9 (−0.3; 2.1)
FU3-FU2	0.2 (−0.9; 1.2)	−0.2 (−1.4; 1.0)	0.8 (−1.2; 2.8)
FU4-FU3	−1.5 (−2.5; −0.5)	−0.7 (−1.8; 0.5)	−0.4 (−1.6; 0.8)
FU5-FU4	0.6 (−1.0; 2.2)	−0.7 (−2.5; 1.1)	−0.0 (−2.0; 2.0)
FU4-PREOP	−2.2 (−4.2; −0.2)	1.2 (−0.7; 3.2)	−4.1 (−6.4; −1.8)

**Table 5:** Differences in angles in degrees between C2 and C7 (mean ± SD) for the different follow-up (FU) times (PREOP: preoperative; POSTOP: 1 day postoperatively)

	ACDA	ACDF	ACD
POSTOP-PREOP	−1.4 (−4.8; 2.1)	−0.1 (−3.1; 3.0)	−6.0 (−9.9; −2.1)
FU2-POSTOP	2.2 (−0.7; 5.1)	3.8 (0.9; 6.6)	3.0 (−0.4; 6.3)
FU3-FU2	−0.6 (−3.3; 2.1)	0.2 (−2.0; 2.4)	3.4 (0.1; 6.5)
FU4-FU3	−1.3 (−4.5; 1.9)	1.4 (−2.6; 5.5)	−0.4 (−4.7; 3.9)
FU5-FU4	2.8 (−2.7; 8.3)	−4.1 (−10.8; 2.5)	−3.1 (−9.6; 3.4)
FU4-PREOP	−0.9 (−4.4; 2.5)	7.3 (3.7; 10.9)	1.0 (−4.0; 5.9)

**Table 6:** The pre- and postoperative sagittal curve of the cervical spine defined as lordotic, straight and kyphotic

		Postoperative shape		
		Kyphosis	Straight	Lordosis
Procedure	Preoperative shape			
ACDA	Kyphotic	4	6	1
	Straight	0	15	4
	Lordotic	0	23	0
	Unkown	0	2	0
ACDF	Kyphotic	3	1	2
	Straight	0	12	7
	Lordotic	0	1	17
ACD	Kyphotic	4	1	0
	Straight	0	21	5
	Lordotic	0	12	0
	Unknown	0	1	0

## Discussion

Most radiological studies in relation to arthroplasty focus on ROM and movement of the adjacent levels in comparison to ACDF with plate fixation. A few studies have described the sagittal cervical balance after arthroplasty in comparison ACDF with plate fixation [2, 18, 26]. Retrospective cohorts have also been published [1, 3, 13]. A recent systematic review showed that after ACDA the alignment of the cervical spine tended to become kyphotic [10], which concurred with our results.

This is the first study that evaluated the effect of ACD, ACDF with a stand-alone cage and ACDA on cervical sagittal alignment at both the involved (affected) level and the cervical spine (C2–C7). While ACD is a well-known procedure, it has received little attention in the last 10 years. The research has focused on comparing ACDF with plate fixation with ACDA. This study is unique because of its prospective nature and the comparison of ACDA with ACDF with a stand-alone cage and with ACD.

Two remarkable findings of this study should be mentioned. First, though the angle at the involved level became more lordotic after ACDF and more kyphotic after ACD, it tended to normalize to its former preoperative value at approximately 9-week’s evaluation for ACDF. For ACD the change was only minimal (one degree) and remained locally kyphotic. Moreover, after nearly 1 year, no differences between the three procedures could be detected. Second, global cervical lordosis was not affected by the three different techniques. These findings can be explained by the natural mechanism of the human body to maintain the head in a neutral axis in the horizontal plane optimal for the visiovestibular system and restore sagittal balance. To maintain the global sagittal balance, it seems logical that after a relatively small disturbance at the involved level, it will be locally resolved and affect the whole spine. It should be emphasized that the current investigation has only focused on radiological cervical sagittal balance and the effect of time on both the involved (affected) levels and the global cervical spinal curvature. However, Carreon et al. showed a good correlation of the measurements on the lateral full spine radiographs compared to the dedicated lateral cervical radiographs [8].

Statements about clinical performance or quality of life after the different procedures cannot be made from these results. However, our study clearly shows that the way of performing an anterior cervical discectomy does not affect cervical lordosis in time. Therefore, one might assume that any eventual difference in clinical results should not be attributed to sagittal alignment. Considering the goal of our study, the lack of correlation with clinical outcome is not a weakness of the study but a strength, since the focus on the sagittal balance contributed to a clear interpretation of the results and discussion.

The chosen procedure only seemed to affect the angle of the involved segment to a minor degree. Therefore, the argument of restoring lordosis by increasing it at one involved level for one-level degenerative disease is at least debatable. A limitation of our study is the loss of patients to radiological follow-up resulting in larger standard deviations. However, we feel confident that the findings represent the actual situation since the angle at the surgical level did not change significantly after the second follow-up time and the sagittal angle of C2–C7 at the last follow-up was similar to the preoperative one.

Furthermore, while the study was ongoing, more patients questioned why they should visit the hospital since they could report their outcome measurements at home. Many of them found the radiographic control examinations irrelevant unless symptoms occurred. Therefore, we adapted the protocol. In the literature arguments were found to support this alteration [4]. Furthermore, the primary outcome measure of the trial was clinical outcome. To optimize the participation of the patients, we were willing to facilitate their cooperation.

Not including the C2–C7 SVA, T1 slope or other measurements in our study might be debated. However, since a good correlation was estimated with the Cobb angles [8, 17], and we were interested also in the angle of the involved angle, we decided to measure the C2–C7 angle. Furthermore, attention was given to changes within groups of patients. Due to the kind of procedure, we did not assume that a clinically significant translation within the cervical spine would occur since posterior elements remained untouched and the disruption of the anterior part was minimal. Otherwise, measurement of the C2–C7 SVA would be more appropriate [20, 22].

Our study shows that the most important changes to cervical alignment took place 1 day after the surgery and in the immediate weeks thereafter. From FU2 (approximately 9 weeks), changes did not occur anymore in the local angle or in the global cervical sagittal alignment. The major strength of the study is the design, facilitating the formation of three groups, with comparable radiological baseline characteristics. This made the study unique. The high inter-rater reliability also contributed to the strength of this study.

In conclusion, at longer follow-up sagittal cervical alignment was not affected by the procedure for cervical anterior discectomy. Despite the initial increase or decrease of lordosis at the involved level, the tendency developed to restore local cervical alignment to the preoperative situation. This could be interpreted as a natural inborn mechanism to restore a long-standing situation to which the body has been accustomed. Restoring local cervical lordosis as an argument to promote a certain procedure for a single level cervical degenerative disc disease is at least debatable.
